# American Medical Association

**Published:** 1875-05-15

**Authors:** 


### Editorial Department.

### AMERICAN MEDICAL ASSOCIATION.

THE Twenty-Sixth Annual Meeting of this Association, was held in Louisville, Ky., commencing on the morning of May 4th, 1875, and ending at noon on the 7th. There was present a more general representation of the profession in all parts of the country, than for several years before. The whole number was not less than five hundred, embracing in the list many of the oldest and most distinguished members of the profession in this country. Dr. Botsford, of New Brunswick, president of the Canadian Medical Association, was also present, and occupied a seat on the platform.

The arrangements for the general meetings were good; those for the sections better than in some former years, but not as perfect as they should be. On the whole, however, the necessary arrangements for the working of the Association were good, and the meeting throughout was characterized by good order, cordiality of feeling, and faithful devotion to business.

The number and character of the papers read and discussed in the several sections, were better than the average of former years, while the addresses of Drs. Austin Flint, S. M. Moore, Wm. H. Byford, and H. J. Bowditch, as presidents of the several sections, and the volunteer paper of Dr. Gross, all read in the general sessions of the Association, were full of interest and instruction. Outside of the proper business of the Association, the social reception at the Galt House, and the numerous entertainments furnished by the profession and citizens of Louisville, were most ample in number, and elegant in character. The absence of intoxicating beverages from nearly all the receptions and entertainments provided, was noted with pleasure, and most highly approved by all present.

The address of Dr. Wm. K. Bowling, of Nashville, as president of the Association, was excellent in matter, and appropriate in style, and was listened to with great interest.

He recalled the original objects for which the Association was organized, noted the progress thus far made, the obstacles to be overcome, and the most efficient means for their removal.

We especially call the attention of our readers to the following, which constituted the closing part of his address:

"The question returns to us—What can the Association now do, in its early manhood, honestly, toward redeeming implied pledges in its infancy? Much, if it have nerve or backbone; nothing, if these be absent! The plan is simple, as all plans are that succeed. Logic and truth are simple, but without nerve the whole moral world is like an empty sack, utterly incapable of standing erect. The barrier to success has been removed by the abolition of school representation, as such, and reducing the whole body to "lay" members. It was to secure the success of the plan to be proposed, to elevate the standard of medical education, that the resolution was introduced at Nashville in 1857, to remove the schools from the Association.

"In the arbitrary numbering of the objects for the promotion of which this body was created, that of number eight is declared to be 'To take cognizance of the common interest of the medical profession in every part of the United States.' A very comprehensive power, assumed in the beginning, and never denied in all these years, will not be questioned now, when the moral frown of the Association would be fatal to whoever, or whatever, connected with medicine, should oppose the grand and benevolent objects that lie at its foundation. In taking cognizance of the common interest of the medical profession in every part of the United States, it must go back upon itself, and acknowledge its recreancy to the high objects of the fathers, who wore away their lives in an unswerving devotion to it, not to exercise the sum total of its legal and moral force in securing a higher standard of medical education in this country than existed at the time of its inauguration.

"Therefore, let it be solemnly resolved by this meeting, that it shall be regarded as derogatory to the character of any physician, in any part of the United States, to take under his care, as a student of medicine, any one who cannot exhibit evidence of having taken a degree in a regularly chartered college, or a certificate of qualifications necessary to become a student of medicine, from a board of examiners appointed for that purpose by the American Medical Association. This will do the work.

"Territories and new States, in a country like ours, in a formative state, will provide themselves with medical helps in the mode we have described, which, existing outside of this body, and independent of it, will occasion it no concern whatever; nor would the schools suffer pecuniary loss under this rule. When it was generally known, as it soon would be, young men desiring to enter the profession would earnestly devote themselves to the duties of preparation, nor relax their efforts till possessed of the degree or the certificate.

"Again, many educated young men under this rule, would turn their attention to medicine, whose votaries were to consist of their peers, who, under the existing rule, would not risk its leveling influences. Let the doctorate imply something more than 'two full courses of lectures, the last of which in this institution.' Besides, it would give the college an ample excuse for not receiving every uneducated lazy dolt who desired to make a living under false pretences.

"There is nothing really binding in the rule suggested. The only power in the matter is, the great moral weight of the Association. It enacts nothing, but simply asserts what every member of it knows to be right. After a few years, such a certificate of the examining board, or evidence of a college degree, might be declared necessary in order to enable an applicant for membership in this body to secure admission; for surely it is the common privilege of all organizations to judge of the qualifications of their own members. Then will the certificate of membership here pass the holder anywhere as a gentleman and scholar.

"It is precisely in this way that the medical department of the army and navy are purified. The adoption of this addition to the code of ethics, would furnish medical gentlemen an excuse for getting rid of applicants for office study, whose preliminary education they know to be defective, and whose relations they would dislike to offend by saying so.

"Neither would this rule exclude any one from being a doctor. In a vigorous republic there will always spring up men who, by genius and long self-training, literally hew their way to greatness in all of the professions, while many more will pass through colleges, winning all their honors, to shrink into insignificance, and pass through the world unknowing and unknown. For the former heaven has made ample provisions, and stamped them as the nobility of nature, whom this body can neither depress nor elevate—nay, nor could an association of angels.

"Under the old plan, as under the new, the schools must furnish the Association with delegates; but under the new the delegate was passed upon and accepted before he was medically born. In this latter case the association begins *de novo*, with the beginning. It is present at both ends of the line. In the former case it proposed to arrange the plumage of another party's chicken, to groom the steed of another. The proposition was arrogant, and excited opposition and clamor. The chicken from the prairie and the steed from the desert, captured by the schools, were theirs, and they naturally threw about them the ægis of protection. Examining boards, after two courses of lectures, were boards born out of time, and their tender mercy to the callow brood was not to be trusted and the preposterous suggestion was scouted. The new plan is a medical sandwich. The schools are a slice of ham between an upper and lower layer of association bread. It must be clear to every thinking mind that the plan, while eminently just and proper, places medical education in the United States where it ought to be, with everything else pertaining to the profession, in the power of the American Medical Association. Medical education, *per se*, will take care of itself, the emulation of the schools being altogether sufficient for the maintenance of its great interest. It is the preliminary education of those who would enter the profession that must be looked to. It must be everywhere known that no medical college, nor any other contrivance short of the fiat of the Almighty, can make a physician out of an uneducated man, that medicine is the cap-sheaf of all knowledge, and the belief that prevails that it can exist in the absence of its legitimate supports is only equaled by that which "materializes" the spirits of the departed before the resurrection.

"God opened the Red Sea to the leader who was skilled in all the learning of the most learned people in existence. The greatest body of water is the lowest, and the foundation of the redemption of the world was confided to the lowly, still, when the glad tidings were to be carried to the learned nations, a profound logician and a philosopher received the appointment; and all, and therefore the greatest, of theological upheavals that have since convulsed the world trace their origin to brains of the most elaborate culture. The god-like Clay, in his speech to his old neighbors, upon retiring from the Senate of the United States, among the obstructions in the path of his early life, alludes feelingly to his 'imperfect education.' Drake, on whose triumphant theatre we are, and whose mighty spirit hovers about it like the aroma of the broken vase, would not go to Europe in his old age, being ashamed of his want of knowledge; and he, whose great soul conceived and planned this organization, and whose eloquent pleadings have held it to its moorings, amid storm and sunshine, through a generation, in a heroic struggle to secure a higher educational plane for his beloved profession, declared that a defective early training had met and abashed him at every turn in his professional life."

